# Neuroimaging article reexecution and reproduction assessment system

**DOI:** 10.3389/fninf.2024.1376022

**Published:** 2024-07-22

**Authors:** Horea-Ioan Ioanas, Austin Macdonald, Yaroslav O. Halchenko

**Affiliations:** Center for Open Neuroscience, Department of Psychological and Brain Sciences, Dartmouth College, Hanover, NH, United States

**Keywords:** reproducibility, reexecutable workflows, fMRI, neuroscience, FOSS (free and open-source software), optogeneitcs, automation, publishing technologies

## Abstract

The value of research articles is increasingly contingent on complex data analysis results which substantiate their claims. Compared to data production, data analysis more readily lends itself to a higher standard of transparency and repeated operator-independent execution. This higher standard can be approached via fully reexecutable research outputs, which contain the entire instruction set for automatic end-to-end generation of an entire article from the earliest feasible provenance point. In this study, we make use of a peer-reviewed neuroimaging article which provides complete but fragile reexecution instructions, as a starting point to draft a new reexecution system which is both robust and portable. We render this system modular as a core design aspect, so that reexecutable article code, data, and environment specifications could potentially be substituted or adapted. In conjunction with this system, which forms the demonstrative product of this study, we detail the core challenges with full article reexecution and specify a number of best practices which permitted us to mitigate them. We further show how the capabilities of our system can subsequently be used to provide reproducibility assessments, both via simple statistical metrics and by visually highlighting divergent elements for human inspection. We argue that fully reexecutable articles are thus a feasible best practice, which can greatly enhance the understanding of data analysis variability and the trust in results. Lastly, we comment at length on the outlook for reexecutable research outputs and encourage re-use and derivation of the system produced herein.

## 1 Background

### 1.1 Reexecutable research

Independent verification of published results is a crucial step for establishing and maintaining trust in shared scientific understanding (Ioannidis, 2005; Open Science Collaboration, 2015). The property of a research workflow to automatically produce an output—analogous, even if incoherent, with the original—based on the same input data and same instruction set is known as reexecutability. This property, though conceptually simple, has remained largely unexplored as a distinct phenomenon in the broader sphere of “research reproducibility.” The core distinction between reexecutability and reproducibility, is that the latter refers to obtaining consistent results when re-testing the same phenomenon (National Academies of Sciences, Engineering, and Medicine, 2019), while the former refers to being able to obtain *any* results, automatically, while re-using the same data and instructions. While the scope of *reexecution* is thus much narrower than that of *reproduction*, it constitutes a more well-defined and therefore tractable issue in improving the quality and sustainability of research. In all cases, reexecutability increases the feasibility of reproduction assessments, as it enables high-iteration re-testing of whatever parts of a study are automated. Further, in the case of complex analysis processes with vast parameter spaces, reexecutability is a prerequisite for detailed reproducibility assessments. Lastly, reexecution constitutes a capability in and of itself, with ample utility in education, training, and resource reuse for novel research purposes (colloquially, “hacking”)—which may accrue even in the absence of accurate result reproduction.

Free and Open Source Software (Stallman, 2002) has significantly permeated the world of research, and it is presently not uncommon for researchers to publish part of their data analysis instructions under free and open licenses. However, such analysis instructions are commonly disconnected from the research output document, which is manually constructed from static inputs. Notably, without fully reexecutable instructions, data analysis outputs and the positive claims which they support are not verifiably linked to the methods which generate them.

Reexecutability is an emergent topic in research, with a few extant efforts attempting to provide solutions and tackle associated challenges. Such efforts stem both from journals and independent researchers interested in the capabilities which reexecutable research processes offer to the ongoing development of their work. Among these, an effort by the eLife journal (Maciocci et al., 2019) provides dynamic article figures based on the top-most data processing output and executable code conforming to journal standards. NeuroLibre (Karakuzu et al., 2022) provides a Jupyter Notebook based online platform for publishing executable books along with a selection of related assets, namely code, data, and a reexecution runtime. Jupyter Notebooks are also used independently of journal support, yet such usage is indicative of a focus on interactivity for top-most analysis steps rather than full reexecution, and characterized by a widespread lack of either data or software dependency specification (Samuel and Mietchen, 2024). Independent researcher efforts at creating reexecution systems offer more comprehensive and flexible solutions, yet remain constrained in scope and generalizability. For example, they may provide reference implementations which are either applied to comparatively simple analysis processes (Dar et al., 2019) or tackle complex processes, but assume environment management capabilities which may not be widespread (Ioanas and Rudin, 2018).

In order to optimally leverage extant efforts pertaining to full article reexecution and in order to test reexecutability in the face of high task complexity, we have selected a novel neuroimaging study, identified as OPFVTA (OPtogenetic Functional imaging of Ventral Tegmental Area projections; Ioanas et al., 2022). The 2022 article is accompanied by a programmatic workflow via which it can be fully regenerated based solely on raw data, data analysis instructions, and the natural-language manuscript text—and which is initiated via a simple executable script in the ubiquitous GNU Bash (Ramey, 1994) command language. The reexecution process in this effort relies on an emerging infrastructure approach, RepSeP (Ioanas and Rudin, 2018), also in use by other articles, thus providing a larger scope for conclusions that can be drawn from its study.

### 1.2 Data analysis

One of the hallmarks of scientific data analysis is its intricacy—resulting from the manifold confounds which need to be accounted for, as well as from the breadth of questions which researchers may want to address. Data analysis can be subdivided into *data preprocessing* and *data evaluation*. The former consists of data cleaning, reformatting, standardization, and sundry processes which aim to make data suitable for evaluation. Data evaluation consists of various types of statistical modeling, commonly applied in sequence at various hierarchical steps.

The OPFVTA article, which this study uses as an example, primarily studies effective connectivity, which is resolved via stimulus-evoked neuroimaging analysis. The stimulus-evoked paradigm is widespread across the field of neuroimaging, and thus the data analysis workflow (both in terms of *data processing* and *data evaluation*) provides significant analogy to numerous other studies. The data evaluation step for this sort of study is subdivided into “level one” (i.e., within-subject) analysis, and “level two” (i.e., across-subject) analysis, with the results of the latter being further reusable for higher-level analyzes (Friston et al., 1995). In the simplest terms, these steps represent iterative applications of General Linear Modeling (GLM), at increasingly higher orders of abstraction.

Computationally, in the case of the OPFVTA article as well as the general case, the various data analysis workflow steps are sharply distinguished by their time cost. By far the most expensive element is a substage of data preprocessing known as registration. This process commonly relies on iterative gradient descent and can additionally require high-density sampling depending on the feature density of the data. The second most costly step is the first-level GLM, as it scales linearly with the number of voxels modeled individually for each subject and session depending on whether or not region of interest masks are used, this number can extend to all voxels in the brain.

The impact of these time costs on reexecution is that rapid-feedback development and debugging can be stifled if the reexecution is monolithic. While ascertaining the effect of changes in registration instructions on the final result unavoidably necessitate the reexecution of registration and all subsequent steps—editing natural-language commentary in the article text, or adapting figure styles, should not. To this end the reference article employs a hierarchical Bash-script structure, consisting of two steps. The first step, consisting in data preprocessing and all data evaluation steps which operate in voxel space, is handled by one dedicated sub-script. The second step handles document-specific element generation, i.e., inline statistics, figure, and TeX-based article generation. The nomenclature to distinguish these two phases introduced by the authors is “low-iteration” and “high-iteration,” respectively (Ioanas and Rudin, 2018).

Analysis dependency tracking—i.e., monitoring whether files required for the next hierarchical step have changed, and thus whether that step needs to be reexecuted—is handled for the high-iteration analysis script via the RepSeP infrastructure, but not for the low-iteration script.

### 1.3 Software dependency management

Beyond the hierarchically chained data dependencies, which can be considered internal to the study workflow, any data analysis workflow has additional dependencies in the form of software. This refers to the computational tools leveraged by the workflow—which, given the diversity of research applications, may encompass numerous pieces of software. Additionally, individual software dependencies commonly come with their own software dependencies, which may in turn have further dependencies, and so on. The resulting network of prerequisites is known as a “dependency graph,” and its resolution is commonly handled by a package manager.

The OPFVTA article in its original form relies on Portage (Amadio and Xu, 2016), the package manager of the Gentoo Linux distribution. This package manager offers integration across programming languages, source-based package installation, and wide-ranging support for neuroscience software (Ioanas et al., 2017). As such, the dependencies of the target article itself are summarized in a standardized format, which is called an ebuild—as if it were any other piece of software. This format is analogous to the format used to specify dependencies at all further hierarchical levels in the dependency tree. This affords a homogeneous environment for dependency resolution, as specified by the Package Manager Standard (Bennett et al., 2017). Additionally, the reference article contextualizes its raw data resource as a dependency, integrating data provision in the same network as software provision.

While the top-level ebuild (i.e., the direct software dependency requirements of the workflow) is included in the article repository and distributed alongside it, the ebuilds which specify dependencies further down the tree are all distributed via separate repositories. These repositories are version controlled, meaning that their state at any time point is documented, and they can thus be restored to represent the environment as it would have been generated at any point in the past.

### 1.4 Software dependencies

The aforementioned infrastructure is relied upon to provide a full set of widely adopted neuroimaging tools, including but not limited to ANTs (Avants et al., 2011), nipype (Gorgolewski et al., 2011), FSL (Jenkinson et al., 2012), AFNI (Cox, 1996), and nilearn (Abraham et al., 2014). Nipype in particular provides workflow management tools, rendering the individual sub-steps of the data analysis process open to introspection and isolated reexecution. Additionally, the OPFVTA study employs a higher-level workflow package, SAMRI (Ioanas et al., 2019a, 2021), which provides workflows optimized for the preprocessing and evaluation of animal neuroimaging data.

### 1.5 Containers

Operating system virtualization is a process whereby an ephemeral “guest” environment is started in and may be reused between persistent “host” systems. Virtual machines (VMs), as these “guest” environments are called, can thus provide users with environments tailored to a workflow, while eschewing the need to otherwise (e.g., manually or via a package manager) provide the tools it requires. Once running, VMs are self-contained and isolated from the host, also eliminating the risk of unwanted persistent changes being made to the host environment. Perhaps the most important benefit of virtual isolation is significantly improved security, allowing system administrators to safely grant users relatively unrestricted access to large-scale computational capabilities. However, VMs can also help mitigate issues arising from package updates by locking a specific dependency resolution state which is known to work as required by a workflow, and distributing that instead of a top-level dependency specification which might resolve differently across time.

Modern advances in container technology allow the provision of the core benefits of system virtualization, but lighten the associated overhead by making limited use of the host system, specifically the hypervisor. Container technology is widespread in industry applications, and many container images are made available via public image repositories. While container technology has gained significant popularity specifically via the Docker toolset, it refers to an overarching effort by numerous organizations, now best represented via a Linux Foundation project, the “Open Container Initiative” (OCI). The OCI governing body has produced an open specification for containers, which can be used by various container runtimes and toolsets. Generally, OCI-compliant container images can be executed analogously with Docker, Podman, or other OCI compliant tools.

While OCI images are nearly ubiquitous in the software industry, Singularity (recently renamed to Apptainer) is a toolset that was developed specifically for high-performance computing (HPC) and tailored to research environments. A significant adaptation of Singularity to HPC environments is its capability to run without root privileges. However, recent advances in container technology have provided similar capabilities. Further, Singularity permits the conversion of OCI images into Singularity images, and recent versions of Apptainer have also added support for natively running OCI containers—thus making reuse of images between the two technologies increasingly convenient.

Container technology thus represents a solution to providing stable reusable environments for complex processes, such as the automatic generation of research articles. In particular, containers provide a convenient way of making advanced package management solutions—as seen in the original OPFVTA article—available to users which may lack them on their host systems.

### 1.6 Hardware requirements

The reproduction of computational analyaes may require specific hardware availability. The OPFVTA study uses processing instructions that can be executed on Central Processing Units (CPUs) and do not require access to a Graphical processing unit (GPU). Resource usage with respect to CPU cores and Random Access Memory (RAM) are scaled dynamically by the workflow, with a system using an i7-8550U CPU and having 16 GB of RAM being the lower bounds of resources for which usage was documented. In cases of lower resource availability the workflow adapts by reducing the parallel processing of individual measurement time series.

## 2 Results

### 2.1 Repository

The repository constituting the output of our work is published openly and with version control based on Git (Torvalds and Hamano, 2024) via GitHub, a social coding platform (Ioanas et al., 2024a) and via Gin, an academic code and data sharing platform (Ioanas et al., 2024b). The most up to date instructions for accessing reexecuting our work (the original as well as this article) are found in the README.md file on the repository. While the key focus on reexecution means that the software internal to the article workflows is provided via containers, software requirements remain for fetching the software, data, and containers themselves. These include, prominently, Git, DataLad (Halchenko et al., 2021a), and a container management system (Docker, Podman, or Singularity).

**Figure 1:**
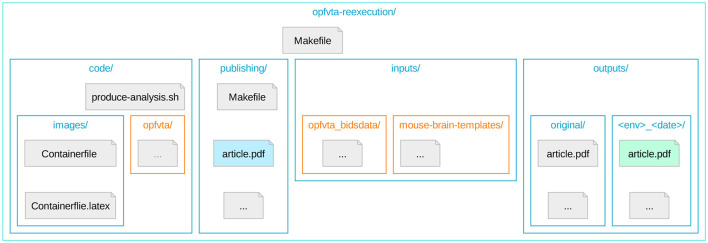
The directory topology of the reexecution repository (Ioanas et al., 2024a), highlighting Git submodules. Depicted is the directory tree topology of the repository coordinating OPFVTA reexecution. Nested directories are represented by nested boxes, and Git submodules are highlighted in orange. The article reexecution PDF results are highlighted in light green, and the PDF of the resulting meta-article (i.e., this article) is highlighted in light blue.

In order to prevent resource duplication and divergence, and to improve the modularity in view of potential re-use of this system, we have bundled access to all elements of our work into a parent repository. This structure (Figure 1) uses Git submodules for referencing individual elements relevant for the workflow, and DataLad in order to permit Git integration with data resources.

These submodules include the original article, the raw data it operates on, and a reference mouse brain templates package. Additionally, the top-level repository directly tracks the code required to coordinate the OPFVTA article reexecution and subsequent generation of *this* article. The code unique to the reexecution framework consists of container image generation and container execution instructions, as well as a Make file and is tracked directly via Git.

This repository structure enhances the original reference article by directly linking the data at the repository level, as opposed to relying on its installation via a package manager. The OPFVTA article source code itself is not duplicated as part of our work, but handled as a Git submodule, with all proposed improvements being contributed to the original upstream repository. The layout constructed for this study thus provides robust provenance tracking and constitutes an implementation of the YODA principles (a recursive acronym for “YODAs Organigram on Data Analysis”; Hanke et al., 2018).

**Figure 2:**
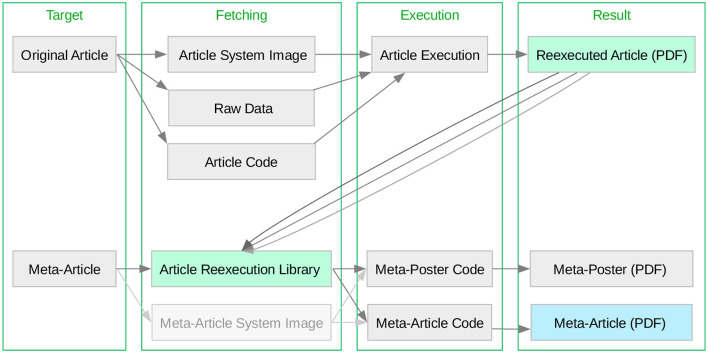
The reexecution system encompasses both the Original Article and Meta-Article as independent make targets. Depicted is the reexecution system workflow, with two reexecution entry points, the “Original Article” and the “Meta-Article” (i.e., *this* article, which also performs the reproduction assessment). Notably, for the generation of the meta-article, the Original Article can be executed, or not—the meta-article will dynamically include all reexecution results which are published, as well as all which are locally produced. The article reexecution PDF results are highlighted in light green, and the PDF of the resulting meta-article (i.e., this article) is highlighted in light blue. Optional nodes (such as fetching a container image for meta-article reexecution) are faded gray.

The Make system (Figure 2) is structured into a top-level Makefile, which can be used for container image regeneration and upload, article reexecution in a containerized environment, and meta-article production. There are independent entry points for both *this* and the original article making both articles reexecutable. Versioning of the original article reexecution is done via file names (as seen in the outputs/ subdirectories of Figure 1) in order to preserve shell accessibility to what are equivalent resources. Versioning of the meta-article is handled via Git, so that the most recent version of the work is unambiguously exposed.

The meta-article targets redirect to a Makefile in the article/ subdirectory, which contains this document's human-readable text in T_E_X format, alongside scripts for generating dynamical elements based on the reexecution results seen in the outputs/ directory. The original article reexecution is provided by two alternative targets, using either the Open Container Initiative standard, or Singularity. Both original article reexecution targets wrap the produce_analysis.sh script, which is a thin compatibility layer accessing the Make system of the original article. This alternative is introduced in order to assess feasibility as well as potential variability across virtualization infrastructures.

### 2.2 Resource refinement

As a notable step in our article reproduction effort, we have updated resources previously only available as tarballs (i.e., compressed tar archives), to DataLad. This refinement affords both the possibility to cherry-pick only required data files from the data archive (as opposed to requiring a full archive download), as well as more fine-grained version tracking capabilities. In particular, our work encompassed a re-write of the Mouse Brain Templates package (Ioanas et al., 2019d) Make system. In its new release (Ioanas et al., 2019c), developed as part of this study, Mouse Brain Templates now publishes tarballs, as well as DataLad-accessible unarchived individual template files.

### 2.3 Best practice guidelines

As part of this work we have contributed substantial changes to the original OPFVTA repository, based on which we formulate a number of best practice guidelines, highly relevant in the production of reexecutable research outputs.

#### 2.3.1 Errors should be fatal more often than not

By default, programs written in the majority of languages (including e.g., Python and C) will exit immediately when running into an unexpected operation. The POSIX shell and other similar or derived shells, such as Bash and Zsh, behave differently. Their default is to continue with execution of the next scripted command, and only exit with a non-zero code when the list of commands is exhausted or the exit command is called explicitly. As a result, an execution of the script could continue for hours before it fails, and the original error message might be lost in the flood of output, making it hard or impossible to localize and address the original problem. This behavior can be mitigated by prepending set -e to the respective shell script, which changes the default behavior so that execution is stopped as soon as a command exits with an error code. Additionally, shell scripts treat undefined variables as a variable having an empty value, with empty values causing no errors. This can lead to numerous ill-defined behaviors, including a command such as rm -rf “$PREFIX/” removing all files on the system if PREFIX is not defined. This can be addressed by prepending set -u so that an error is raised and execution is stopped as soon as an undefined variable is referenced. To summarize, we recommend including set -eu at the top of every shell script to guarantee it exits as soon as any command fails or an undefined variable is encountered. This is in line with the “Fail Early” principle advocated in the ReproNim Reproducible Basics Module (Halchenko et al., 2021b).

#### 2.3.2 Avoid assuming or hard-coding absolute paths to resources

Ensuring layout compatibility in different article reexecution environments is contingent on processes being able to find required code or data. Absolute paths, which are hard-coded into scripts, are likely to not exist anywhere but the original execution environment, rendering the scripts non-portable. This problem is best avoided by adhering to YODA principle (Hanke et al., 2018) of being able to reference all needed resources (data, scripts, container images, etc.) *under* the study directory. Use of relative paths within the study scripts consequently improve their portability. Paths to external resources (scratch directories or reusable resources such as atlases) should additionally be parameterized so that they can be controlled via command line options or environment variables.

#### 2.3.3 Avoid assuming a directory context for execution

As previously recommended, resources may be linked via relative paths, which are resolved based on their hierarchical location with the respect to the execution base path. However, scripts could be executed from various locations and not necessarily from the location of the script, thus rendering relative paths fragile. A good way of making script execution more robust is ensuring that they set base execution directories to their respective parent directories. This can be accomplished in POSIX shell scripts by prepending cd ~$(dirname ~$0~)~.

#### 2.3.4 Workflow granularity greatly benefits efficiency

The high time cost of executing a full analysis workflow given contemporary research complexity and technical capabilities makes debugging errors very time-consuming. Ideally, it should not be necessary to reexecute the entire workflow for every potentially resolved error. It is thus beneficial to segment the workflow into self-contained steps, which can be executed and inspected independently. Workflows should as a minimum separate such large steps as preprocessing, individual levels of analysis (e.g., per-subject vs. whole-population), and article generation. One way to integrate such steps is to formulate a workflow which automatically checks for the presence of results from prior stages, and, if present, proceeds to the next stage without triggering prior processes. This property is known as itempotence and is again advocated by the YODA principles, and implemented in this article via both the Make system, as well as internally by the original article's usage of NiPype.

#### 2.3.5 Container image size should be kept small

Due to a lack of persistency, addressing issues in container images requires an often time-consuming rebuild process. One way to mitigate this is to make containers as small as possible. In particular, when using containers, it is advisable to *not* provide *data* via a package manager or via manual download inside the build script. Instead, data provisioning should be handled outside of the container image and resources should be bind-mounted after download to a persistent location on the host machine.

#### 2.3.6 Resources should be bundled into a superdataset

As external resources might change, it is beneficial to use data version control system, such as git-annex and DataLad. The git submodule mechanism permits bundling multiple repositories with clear provenance and versioning information, thus following the modularity principle promoted by YODA. Moreover, git-annex supports multiple data sources and data integrity verification, thus increasing the reliability of a resource in view of providers potentially removing its availability.

#### 2.3.7 Containers should fit the scope of the underlying workflow steps

In order to constrain the workload of rebuilding a container image, it is advisable to not create a bundled container image for sufficiently self-contained substeps of the workflow. For example, as seen in this study, the article reexecution container image should be distinct from container images required for producing a summary meta-article. Conversely, if sub-steps share toolkit requirements, containers can be re-used between different steps by leveraging different *entry points* to the same target.

#### 2.3.8 Do not write debug-relevant data inside the container

Debug-relevant data, such as intermediary data processing steps and debugging logs should not be deleted by the workflow or written to an ephemeral location inside the container, but should instead be written to persistent storage. When using some container technologies, such as Docker, files written to hard-coded paths will disappear once the container is removed. As numerous workflow files beyond the main data output may be relevant for debugging, they should not be lost. In order to achieve this, intermediary and debugging outputs should be written to paths which are bind-mounted to persistent directories on the parent system, from which they can be freely inspected.

#### 2.3.9 Scratch directories should be parameterized

Complex workflows commonly generate large amounts of scratch data—intermediary data processing steps, whose main utility is being read by subsequent steps or consulted for debugging. If these data are written to the same hard-coded path on the host system, multiple reexecutions will lead to race conditions, compromising one or multiple instances of the process. This can be avoided by parameterizing the path and/or setting a default value based on a unique string (e.g., generated from the timestamp). When using containers, this should be done at the container instantiation level, as the relevant path for such potential conflicts is the path on the parent system, and not the path inside the container.

#### 2.3.10 Dependency versions inside container environments should be frozen as soon as feasible

The need for full image rebuilding means that assuring consistent functionality in view of frequent updates is more difficult for containers than interactively managed environments. This is compounded by the frequent and often API-breaking releases of many scientific software packages. While dependency version freezing is not without cost in terms of assuring continued real-life functionality for an article, it can aid stable re-execution if this is done as soon as all required processing capabilities are provided. How this is accomplished differs greatly based on the package manager used inside the container. Gentoo's Portage package manager allows freezing versions both explicitly, or—as done in this study—by checking out a specific commit of the dependency tree, in view of which the package manager will resolve the same versions. Other distributions (such as Debian and Neurodebian), or language-specific package managers (such as Python's pip), provide analogous functionality, via e.g., nd_freeze or pip freeze, respectively.

### 2.4 Reproduction quality

As a top-level view of reexecution results we have produced a simple infrastructure to analyze reproduction quality. This provides both quality control for successful reexecution as well as a showcase of how automatic article reexecutability can be leveraged to evaluate *reproducibility* at a glance.

**Figure 3:**
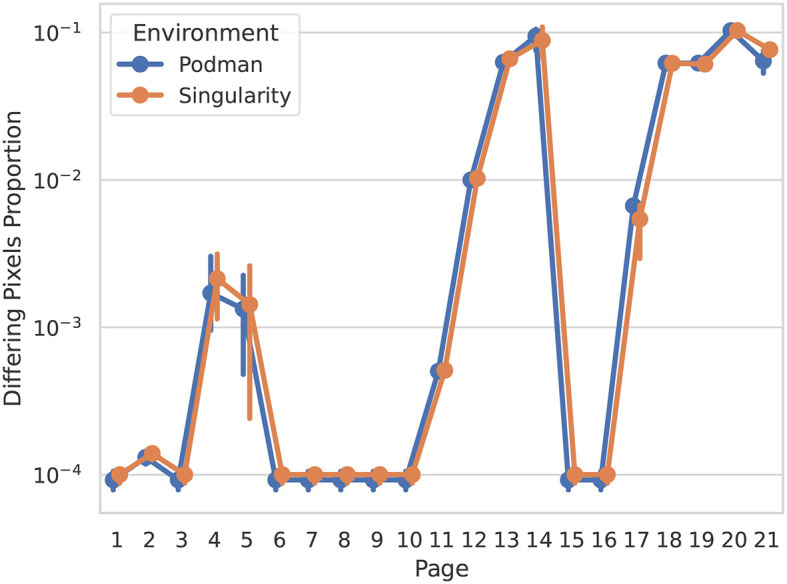
Page-wise pixel differences between the Historical Manuscript Record and new reexecution system outputs gauge overall reproduction fidelity, and highlight pages with noteworthy differences. Depicted are rasterized document differences, weighted 1 for changes in any pixel color channel, and rounded to four decimal points. Error bars represent the 95th percentile confidence interval. Colors indicate the reproduction environment, Singularity, and Podman—the latter being an OCI implementation analogous to Docker.

For this purpose we compare the difference between the Historical Manuscript Record—a product of the original executable article generation—and multiple results generated via the new reexecution system. Reproduction differences between the article versions are extracted by evaluating rasterized page-wise PDF differences (Figure 3).

**Figure 4:**
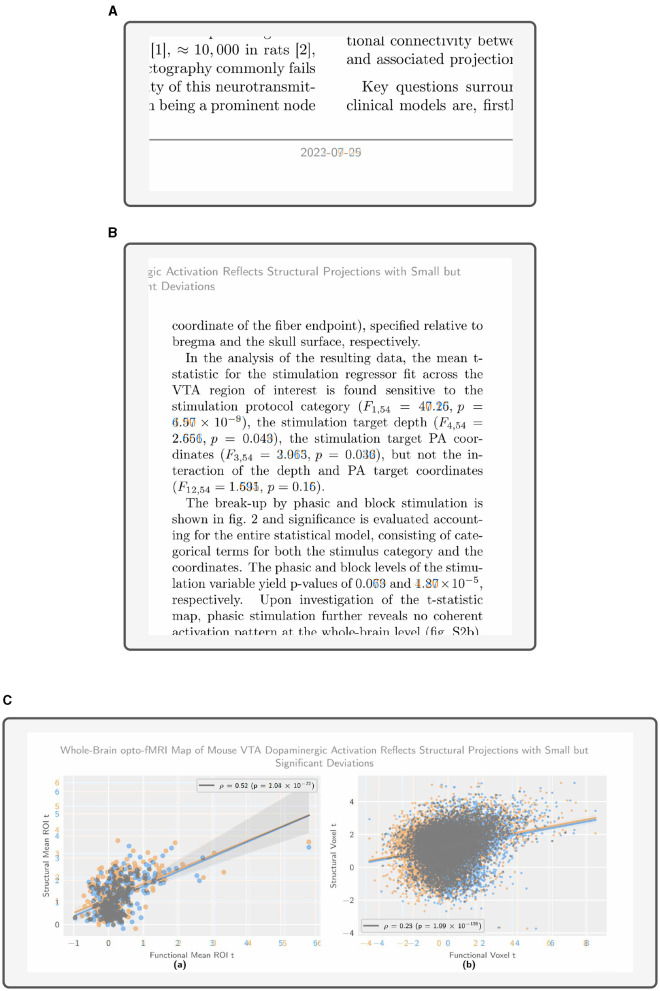
The article differences showcase expected quantitative and metadata variability, while maintaining overall validity of qualitative statements. The figures are extracted from a full article diff, with tinted highlighting (blue for the Historical Manuscript Record, and orange for the new reexecution system result). **(A)** The date change is correctly identified throughout the document, as seen in this example from page 1 of the article. **(B)** Statistical summary values change, but maintain qualitative evaluation brackets with respect to e.g., *p*-value thresholds, as seen in this example from page 4 of the article. **(C)** In regression analysis, data points are highly variable, the slope and significance remain constant, as seen in this example from page 14 of the article.

This overview shows a consistent minimum baseline of differing pixels between reexecutions, around 10^−4^ (i.e., 0.01%), best seen in pages 6 to 10. When examined closely (Figure 4A), this difference corresponds to the modified date of the Historical Manuscript Record (2022-07-25) and the new reexecution system results (2023–...). While otherwise inconsequential, this difference provides a good litmus test for whether the article was indeed reexecuted or simply preserved, and should be expected throughout all comparisons. Throughout other pages we see difference percentages which are broadly consistent across reexecutions and environments, but vary from page to page over almost 2 degrees of magnitude. Upon inspection, more variable but comparatively lower-percentage differences (pages 4 and 5, detail depicted in Figure 4B) are revealed as text differences. This is caused by the target article being fully reexecuted, including the reexecution of inline statistic summaries (e.g., *p* and *F*-values). Higher-percentage differences (detail depicted in Figure 4C) correspond to dynamically generated data figures, in which the high variability of nondeterministic preprocessing results in changes to the majority of figure pixels.

Notably, inspecting these differences reveals a strong coherence at the qualitative evaluation level in spite of high quantitative variability. This coherence manifests in the statements from the original article remaining valid with regard to statistical summaries which emerge from *de novo* data processing (as seen in Figures 4B, C). This is particularly true for *p*-values, the magnitude of which can vary substantially at the lower tail of the distribution without impacting qualitative statements.

Further, we find that text differences are well-localized, as a function of the original article implementing fixed decimal rounding and magnitude notation for statistical outputs (Figure 4). Thus, changes in inline statistic values do not impact text length and do not generally propagate to subsequent lines via word shifts, where they would be recorded as false positives.

## 3 Methods

### 3.1 Data acquisition

No new animal data was recorded. The data forming the substrate for the reproduction analysis was produced by extracting the output article.pdf files from iterative reexecutions of the original article code.

### 3.2 Computing environments

Article reexecution was performed on a Debian 6.1.8-1 (2023-01-29) system using the x86_64 architecture, inside containers handled by Podman version 4.3.1 and Singularity version 3.10.3. Git version 2.39.2 and DataLad version 0.19.2 were used for data and code orchestration. The top-level make targets were executed via Bash version 5.2.15.

### 3.3 Data sources

The raw data for the article was sourced in BIDS form from Zenodo, an open data repository, via the identifier specified by the original publication (Ioanas et al., 2019b). Mouse brain templates were sourced via a Git repository, “Mouse Brain Templates,” which was updated as part of this study to allow individual file fetching (Ioanas et al., 2019c).

## 4 Discussion

In this article and its accompanying source code (Ioanas et al., 2024a) we present an automated workflow for full, end-to-end article reexecution. We generate the full research communication output (including inline statistics, figures, and brain maps) from solely the raw data and automatically executable code. This work substantiates the feasibility of article reexecution as a process, based on a real-life peer-reviewed study example. To this end, we also detail important and transferable principles, and document common pitfalls in creating a reexecution workflow. Lastly, we leverage the capabilities of this reexecution system in order to provide a simple reproducibility assessment, showcasing the relevance of reexecutable research outputs for investigating reproducibility.

### 4.1 Reexecutability

We argue that reexecutability is a core aspect of reliable research output creation. Reexecutability implies that instructions are formulated in such a way that they can be automatically deployed without human operator bias. In contrast to arbitrary reporting standards, the property of reexecutability implicitly guarantees that required instructions are fully recorded.

We demonstrate the feasibility of full research output reexecution by integrating cutting-edge technological capabilities, and publish all resources for open access, inspection, re-use, and adaptation. The article reexecution system which we produced isolates data and original resources, and does not make assumptions about the internal structure of a reexecutable article, and is of course, not domain-specific. Our system initiates article execution via a Bash entry point, meaning it itself is programmatically accessible for integration into higher-order reexecutable research. We demonstrate the feasibility of this by integrating the original article reexecution with the reexecution of the meta-article. Dependency resolution for the original article is provided via an ebuild-style specification present in the original article code. This means that its dependencies are resolved seamlessly with all lower-level dependencies, and could be resolved seamlessly with higher-order dependencies making use of the reexecutable article as a piece of software.

We sharply distinguish between reexecutability and reproducibility. The former refers to the capability of producing an analog research output from the same data through automatic execution of data analysis. The latter refers to the coherence between an analog research output (whether automatically reexecuted or manually recreated) and an original research finding. We further distinguish those two terms from replicability, which describes an identical reproduction of a finding.

### 4.2 Reproducibility

We supplement the reexecution workflow description of this article with a brief demonstration of how it can be used to provide a reproducibility assesment. For this purpose we use a difference computation tool (in computational contexts known simply as “diff”) which summarizes and visually displays mismatches between a historical manuscript record and multiple reexecutions over various environments. Such a process makes mismatches visible at-a-glance throughout the article figures and text, rendering them easy to locate and interpret via human inspection.

Based on these results we lay out a few key findings for further reproducibility assessments. In particular, we notice that figures which directly map output data are highly and to a consistent extent variable across multiple reexecution attempts. However, in as far as such figures are accompanied by statistical evaluations, we find these to be qualitatively consistent. This indicates that reproduction quality is not only reliant on whether or not data processing is deterministic, but also on which aspects of the top-level data the authors seek to highlight. While the above observations describe the current article specifically, we suspect that they may reflect a phenomenon of broader relevance.

In neuroimaging workflows, the most notorious source for non-deterministic data analysis behavior is the registration. This process commonly operates via a random starting point—specified by a seed value—and iterates according to a gradient descent algorithm. While the toolkit used by the OPFVTA article allows the specification of a particular seed, this was not done for the Historical Manuscript Record, nor is it a feature commonly used by operators. In light of our results, the question emerges whether or not seed specification should be introduced as a best practice. While a fixed seed would aid in numerical reproducibility, it is possible that a specific seed—whether by coincidence or *ex post facto* selection—may result in anomalous conclusions. It may then be that a stronger finding is one which is statistically robust with respect to preprocessing variability, even if this comes at the cost of compromising numerical replicability. Conversely, it could be argued that reproduction analysis can be better targeted and more concise, if seed values were fixed to universally accepted numbers (analogous to the usage of nothing-up-my-sleeve numbers in cryptography). Additionally, fixed seed values might consolidate quality control, as quality control and data exclusion based on post registration data would be consistent across executions. This is a significant concern, since incidental data distortion is a well-documented phenomenon with registration workflows (Ioanas et al., 2021).

### 4.3 Challenges

For this meta-article we have selected an original neuroimaging article which already published all of the instructions needed to reproduce it in its entirety from raw data and automatically executable instructions. Even in light of this uncommon advantage, setting up a portable reexecution system has proven to be an ample effort.

Difficulties arose primarily due to the instability of the software stack. As researchers become involved in open source software development, it is becoming increasingly common for scientific software to be subjected to frequent interface changes and loss of support for older dependency versions. In this article we propose version-frozen container technology as a mitigation method for such fragility. However, this is not without draw-backs, as it can make introspection more challenging. In view of this, we defined interactive container entry points (make targets), whereby the user may enter the container dedicated to automatic reexecution to inspect and test changes in the environment. Even so, on account of these containers being dedicated to automatic execution, features such as an advanced text processor are missing, and the inclusion of such features may not be ultimately desired.

A more easily surmountable challenge was data management. Whereas the original article strove to integrate all provision of computational requirements with the package manager, the usage of containers made the cost of this all-encompassing solution prohibitive. As such, Git submodules and DataLad were used, providing enhanced functionality for e.g., data version specification, but at the cost of spreading requirements provision over different technologies.

A further and unavoidable challenge consisted in the execution time-cost. While not prohibitive, the time cost not only slows iterative development work, but presages a potential decrease in the feasibility of reexecution given the trend toward larger and larger data. This means that process complexity and experimental data size may need to be evaluated in light of the diminished accessibility to such processes as reexecution.

Lastly, a notable barrier to execution may be produced by hardware requirements. While this is not manifest in the current study, increasingly many processes may require Graphical Processing Unit (GPU) access as a hardware requirement which cannot be virtualised. The handling of such situations would be a significant concern for making the reproduction of studies with specific hardware requirements more broadly accessible.

### 4.4 Outlook

We propose a few key considerations for the further development of article reexecution—though we note that practical reuse of this system might identify promising enhancements better than theoretical analysis.

In particular, we find that reexecutable article debugging in a container environment can be a significant challenge, and one which will only be more severe if such an environment is already implemented in the development phase of an article. In order to provide seamless integration of both flexible development and portable reexecution, we envision a workflow system which, for each analysis step, permits either usage of locally present executables, or entry points to a container. These two approaches may also be integrated by bind-mount overloading of container components with their local counterparts. We implement a version of this concept for the meta-article generation, where the make article target which generates this article will use the local environment, and the make container-article target executes the same code via an entry point to a T_E_X container.

The reproduction quality assessment methods provided in this study serve as a starting point for assessing full article reexecution. We argue that for the reproducibility assessment of a specific article, there is currently no substitute for the human-readable article as the foremost output element, as it most accurately documents all variable elements in the context of the statements they underpin. However, it should be noted that crude pixel-diff comparison, as showcased here, cannot provide automatic evaluation of differences (i.e., determining whether or not statistical thresholds have been crossed)—so machine-readable outputs are necessary for numerical comparisons. There are ongoing efforts, such as NIDM (Maumet et al., 2016), to establish a framework and language for describing numerical results in neuroimaging. This requires custom tooling to export result descriptors in a language aiming to approximate but distinct from human readable commentary, and was not yet implemented in our analysis workflow. There are also *supplementary* outputs which may provide additional capabilities, not in lieu of, but in addition to the article text. The foremost among these—specifically pertaining to neuroimaging—are statistical brain maps. Such supplementary data would not only let studies generate reusable outputs, but would also aid the inspection of the original article. Our workflow produces and records all of the top-level data (statistical maps, data tables, etc.) from which the article extracts elements relevant to its statements. We have uploaded the main statistical map of reexecution results to NeuroVault, and are working to provide a corresponding template for our mouse brain data. Integration between the present reexecutable article system and statistical map libraries is thus a promising endeavor for further development.

Lastly, we highlight the relevance of reexecutable articles for reuse and adaptation. Their key strength is that they can easily be derived based on a reliable starting point with respect to successful process execution. This pertains not only to reuse of reexecutable article code for novel or derived studies, but also reuse for the inspection of specific parameter or data modifications. In view of this we recommend a practical approach to the work described herein (Ioanas et al., 2024a), whereby the parent reexecution system repository can be considered immediately and freely available for inspection, personal exploration, and re-use by the reader.

## Data availability statement

The original contributions presented in this study are included in the article/supplementary material, further inquiries can be directed to the corresponding author.

## Author contributions

H-II: Conceptualization, Data curation, Formal analysis, Investigation, Methodology, Resources, Software, Supervision, Validation, Visualization, Writing – original draft, Writing – review & editing. AM: Conceptualization, Formal analysis, Investigation, Methodology, Resources, Software, Supervision, Validation, Writing – original draft, Writing – review & editing. YH: Conceptualization, Formal analysis, Funding acquisition, Investigation, Methodology, Project administration, Resources, Software, Supervision, Validation, Writing – original draft, Writing – review & editing.
